# Cancer-Preventive Activity of *Argemone mexicana* Linn Leaves and Its Effect on TNF-α and NF-κB Signalling

**DOI:** 10.3390/cancers15235654

**Published:** 2023-11-30

**Authors:** Sunanda Kulshrestha, Anjana Goel, Nikhat J. Siddiqi, Sabiha Fatima, Bechan Sharma

**Affiliations:** 1 Department of Biotechnology, GLA University, Mathura 281406, India; sunanda.gla_phdbio20@gla.ac.in; 2 Department of Biochemistry, College of Science, King Saud University, P.O. Box 22452, Riyadh 11495, Saudi Arabia; 3 Department of Clinical Laboratory Sciences, College of Applied Medical Sciences, King Saud University, Riyadh 11495, Saudi Arabia; sabmehdi@ksu.edu.sa; 4 Department of Biochemistry, Faculty of Science, University of Allahabad, Allahabad 211002, India; bechansharma@gmail.com

**Keywords:** *Argemone mexicana*, cancer preventive, DMBA/TPA, mouse model, skin cancer

## Abstract

**Simple Summary:**

*Argemone mexicana* is a plant with evidence of traditional use for skin ailments including infection, psoriasis and cancer. The ethanolic extract was administered to experimental animals with skin cancer induced by the DMBA/TPA method. Cancer-preventive activity was found in a dose-dependent manner with the best activity in the pretreated 500 mg/kg BW group. The expression of TNF-α and regulation of the NF-κB signalling pathway were also studied and found to be reduced significantly (*p* < 0.001), which helped with the prevention of cancer.

**Abstract:**

Skin cancer is the 5th most common cancer in Western countries with a surge in case occurrences making it a global burden on healthcare systems. The present study aims to evaluate the cancer-preventive activity of an ethanolic extract of *Argemone mexicana* Linn leaves (AML). The DMBA/TPA method was used to induce skin cancer in mice. Experimental animals were divided into three pretreatment groups of 100 mg/kg BW, 250 mg/kg BW, and 500 mg/kg BW of AML extract, and feeding was continued during the induction process. In the fourth group, 500 mg/kg BW AML extract treatment was started along with the cancer induction. The analyses were performed on the basis of the time period of in-tumour induction incidence, haematological parameters, histopathology and augmentation of TNF-α secretion and the NF-κB (p65 subunit) signalling pathway. The AML extract resisted and delayed tumour formation for up to 8 weeks in the 500 mg/kg BW pretreated group as compared to 4 weeks in the negative control group. The tumour burden varied in a dose-dependent manner in the different groups. On the 60th day, a significantly high burden (*p* < 0.001) was observed in the negative control group and the 100 mg/kg BW group. The study was validated by investigating the expression of TNF-α and the p65 subunit of the NF-κB signalling pathway, which were found to be reduced significantly in a dose-dependent manner and significantly reduced (*p* < 0.001) in the 500 mg/kg BW group as compared to negative control group. The 500 mg/kg BW pretreated group was found to have significant results in comparison to the 500 mg/kg BW post-treatment group. The study revealed the effective cancer preventive activity of *Argemone mexicana* Linn leaves (AML) in the mouse model and paved a pathway for molecular approaches which could be explored more in future studies.

## 1. Introduction

Skin cancer is one of the most prominent cancers, which has 2–3 million cases diagnosed in the world in the past 5 years. This makes it the fifth most common cancer in the world, making it a burden on the economy of any country [1]. Skin cancer cases are increasing at an exponential rate because of prolonged exposure to UV light, the use of chemicals in skin-care products, and the prevalence of immune-suppressing lifestyles within the population [2]. The majority of cancers are caused by a genetic predisposition, environmental pollutants like excessive alcohol or tobacco use, and exposure to hazardous chemicals and radiation. These factors can either be internal body processes like spontaneous mutations, hormones, and nutrient metabolism, or they can be external stimuli [3].

The Indian subcontinent is a well-known biological hotspot with a large variety of flora that could be used for treating many diseases and ailments, including cancer. The literature of Ayurveda has suggested many plants which do not appear useful but contain a treasure of phytochemicals including steroids, flavonoids, and saponins, which could be used to treat cancer [4]. 

*Argemone mexicana* Linn (A.M), from the family Paperveraceae, is an exotic wild plant that is toxic in nature with widespread distribution in many tropical and subtropical countries including west Africa, India, and South America [5]. It is a prickly annual herb and has yellow scentless flowers on it. It is an important medicinal plant and is extensively used in Ayurveda, Siddha, Unani and Homeopathic medicines. *Argemone mexicana* is reported to have antimicrobial, antimalarial, larvicidal, nematocidal, antifungal, anticancerous activity, etc. In India, it is used for the treatment of various diseases, using different parts of the plant for treating jaundice, scabies, fungal infections, ulcers, asthma, intestinal infections, skin, cough, and other disease [6,7,8]. In Mali, South Africa, a study also used the plant extract/sap against malaria in a human trial. This study was conducted in clinical trials in Mali and Switzerland [9]. 

Animal studies play a very important role in the validation of such results, such as the study reported by Sharmila and Manoharan, 2011, which used naturally derived rosmarinic acid from the leaves of the rosemary plant and investigated its anticancer potential against DMBA and TPA induced skin cancer in Swiss albino mice [10]. The mouse skin model of multi-stage chemical carcinogenesis represents one of the most established in vivo models for the study of the sequential and stepwise development of tumours, and the former chemicals used are well-known carcinogens. Similar studies have been reported by Manoharan and Selvan (2012) where they have used geraniol, a synthetic product, for the treatment of skin cancer in Swiss albino mice [11]. 

For molecular pathway regulation studies, NF-κB and TNF-a play a critical role in the link between inflammation and cancer through NF-κB’s ability to upregulate tumour-promoting cytokine and survival genes [12]. Wu et al. (2010) defined the combination of TNF-α and NF-κB as an emerging treatment for cancers. The canonical and alternative main NF-κB pathways have been thoroughly described. In the conventional pathway, the inhibitor of κB (IκB) sequesters NF-κB (often a heterodimer of p65 and p50) in the cytoplasm when it is not triggered [13]. A number of upstream signalling pathways target IκB, activating an IκB kinase (IKK) complex made up of one regulatory subunit, NF-κB essential modulator (NEMO, also known as IKKγ), and at least two kinases, IKKα and IKKβ. IKKα and IKKβ have the ability to phosphorylate IκB directly, which causes ubiquitination and 26S proteasome destruction of the protein [14,15]. 

Several studies have been performed in the ethnopharmacological evaluation of anticancer and cytotoxic activity of *Argemone mexicana* leaves and isolated compounds in in vitro cell lines. No animal study has been reported linked to its therapeutic potential against skin cancer. Here, in this study, an attempt has been made to study the cancer-preventive and cancer treatment in DMBA/TPA-induced skin cancer in mice. The study aims to evaluate the effect of an ethanolic extract of *Argemone mexicana* Linn leaves on cancer-prevention and the expression pattern of TNF-α along with modulation of the p65 subunit of the NF-κB signalling pathway in the cancer-induced mouse model. 

## 2. Materials and Methods

### 2.1. Chemicals

DMBA (7,12-dimethylbenz[a]-anthracene) and TPA (*12-O-Tetradecanoylphorbol-13-acetate*) were purchased from Sigma-Aldrich, St. Louis, MO, USA. The standard chemotherapeutic drug, Doxorubicin, was purchased from Pfizer, New York, NY, USA. TNF-α (Kit Catalogue No: ELK9154) and NF-κB (Kit Catalogue No: ELK1387) kits were purchased from ELK Biotechnology, Wuhan, China. 

### 2.2. Sample Collection, Authentication and Preparation of Extracts 

The leaves of *Argemone mexicana* Linn were collected from local areas and villages surrounding GLA University, Mathura, during February and March (coordinates: 27.49° N and 77.69° E). The leaves were authenticated from CIMAP (the Central Institute of Medicinal and Aromatic Plants, Lucknow, India), and the specimen sample was deposited with Voucher no. CIMAP/Bot-pharm/2021/09. A Soxhlet apparatus was used to prepare the ethanolic extract for testing concentrated using a rotary vacuum evaporator (Yamato Scientific Co., Tokyo, Japan) [16]. 

### 2.3. Animal Care and Handling

Swiss Albino mice 4–6 weeks old and ~25 g were purchased from the animal house of NIB (National Institute of Biologicals, Noida, U.P, India) under sanction No. GLAIPR/IAEC/21/PHD/04 and housed in the Animal House Facility, Institute of Pharmaceutical research, GLA University, Mathura, India (No. 1260/PO/Re/09/CPCSEA). The animals were housed in standard husbandry conditions and subjected to an acclimatisation period of 21 days at 22 ± 2 °C temperature and 12 h light/dark cycle. Food was provided in the form of pellets, and water was provided ad libitum. 

### 2.4. Determination of The Non-Toxic Dose of Argemone mexicana Leaves (AML) Extract

For the determination of the maximum non-toxic concentration of *Argemone mexicana* Linn leaves (AML) extract, the animals were divided into 5 groups of 8 animals each. The first group consisted of the animals, which were fed with vehicle 0.01% of DMSO daily for 21 days. From the 2nd to 5th group, animals were fed orally with 100 mg/kg, 250 mg/kg, 500 mg/kg, and 1000 mg/kg BW of AML extract for 3 weeks. Different physical and haematological parameters were measured in the blood of the animals after 21 days of treatment.

### 2.5. Determination of Skin Cancer Preventive Activity of Argemone mexicana Leaves (AML) Extract

#### 2.5.1. Induction of Skin Cancer in Experimental Animals

The two-stage carcinogenesis method using DMBA and TPA was used for cancer induction in mice as per the protocol quoted in Cold Spring Harbour protocol by Kemp et al., 2015 [17] and repeatedly used by researchers including Surien et al. (2022) [18]. The protocol is well known for its localised effect and comparatively less time-consuming formation of cancer papilloma on the skin. The method includes shaving a particular area on the back of mice with an epilator or depilating cream and the application of DMBA and TPA. First, 250 µg/mL stock solution of DMBA was prepared in acetone, and 0.1 mL of this stock was applied twice a week [19], whereas 2.5 µg/mL stock solution of TPA was prepared in acetone and 0.2 mL was applied once a week on the shaved area [20]. The DMBA is the initiator for cancer and TPA is a promoter. DMBA and TPA treatment was stopped in the animals where cancer lesions started appearing, while it was continued in the other animals up to the appearance of cancer lesions on the skin to determine the cancer-preventive activity of the AML extract. 

The animals were divided into 6 groups of 8 animals each:

1st Group: The normal vehicle group was fed with 0.01% DMSO during the experiment and 0.1% acetone was applied on the shaved skin.
2nd Group: The negative control group was fed with 0.01% DMSO, and cancer was induced by the DMBA/TPA method.
3rd Group: The animals were pretreated with 100 mg/kg BW of AML extract for 3 weeks prior to start the application of carcinogens on the skin.
4th Group: The animals were pretreated with 250 mg/kg BW of AML extract for 3 weeks prior to start the application of carcinogens on the skin.
5th Group: The animals were pretreated with 500 mg/kg BW of AML extract for 3 weeks prior to start the application of carcinogens on the skin.
6th Group: The animals were treated with 500 mg/kg BW of AML extract along with the application of carcinogens on the skin. The group was said to be the post-treated (P.T) group.

In all the experimental groups, AML extract feeding was continued until the end of the experiment. 

#### 2.5.2. Effect on Body Weight

The weight of mice was noted every week in all the groups using the weighing scale, and the average % change in weight of mice along the groups was noted up to 60 days.

#### 2.5.3. Effect of AML on Tumour Volume 

The animals were subjected to measurement of tumour volume across the groups using a Vernier calliper. The formula v = $4/3\pi \left(\frac{D1}{2}\right)\left(\frac{D2}{2}\right)(\frac{D3}{2})$ where D1, D2, and D3 are diameters of the tumours (Sharmila and Manoharan, 2012) [10]. The burden was calculated with the formula on the 0th, 30th and 60th day by multiplying tumour volume and the number of tumours on each animal.

#### 2.5.4. Effect on Cancer Induction

After starting the DMBA/TPA treatment, the percentage of animals having cancer lesions was noted at every two-week interval. The treatment was stopped in the animals in which lesions started appearing. In other animals, the DMBA/TPA treatment was continued. The study was conducted until 60 days for the observation of tumour development. Two animals from each group were sacrificed 60 days after starting the experiment. The blood was collected from these animals and used for haematological analysis and the determination of TNF-α and NF-κB signalling. Histopathological analysis of skin tumour tissue was performed. 

#### 2.5.5. Effect on Histopathological Parameters of Skin Cancer Tissue

The tissue samples of skin tumours were cleaned by removing the extra skin. The tumour tissues were fixed in Neutral Buffer Formalin (NBF) and washed in running water overnight to remove the formalin. The tissues were then embedded in paraffin for section cutting using a microtome and then loaded on the slides. The slides were stained in the standard series of haematoxylin and eosin and fixed with Dibutylphthalate Polystyrene Xylene (DPX). The slides were then observed under microscope.

#### 2.5.6. Effect on Haematological Parameters

The blood samples from the sacrificed mice from each group were analysed for RBC, Hb, MCV, HCT, MCH, MCHC, WBC, DLC, and platelets count. 

#### 2.5.7. Effect on Weight and Size of Liver and Spleen

The animals were sacrificed and the weight of the organs including spleen and liver was measured.

#### 2.5.8. Effect on Inflammatory Cytokine TNF-α Concentration in Serum 

The concentrations of TNF-α in the serum of each sacrificed animal was measured using an ELISA kit, (ELK Biotechnology, Wuhan, China) (Catalogue No: ELK9154) as per the manufacturer’s protocol. The plates were pre-coated with the capture antibody. Wells were incubated with 100 µL of serum from each animal and different concentrations of TNF-α standard according to the format and incubated for 90 min. Wells were washed with washing buffer and further incubated with detection antibody. TMB is used as a substrate for the development of colour. The reaction was stopped with stop reagent, and readings were taken at 450 nm.

#### 2.5.9. Effect on p65 Subunit Concentration of NF-κB Signalling Pathway in Serum 

The concentrations of the p65 subunit of the NF-κB signalling pathway were determined in the serum of each animal using an ELISA kit, (ELK Biotechnology, Wuhan, China) (Kit Catalogue No: ELK1387) as per the manufacturer’s protocol. The given protocol was similar to the TNF-α as given above. The readings of the developed colour were taken at 450 nm.

### 2.6. Determination of Skin Cancer Treatment Activity of Argemone mexicana Leaves (AML) Extract

Skin cancer was induced by the DMBA/TPA method in 18 normal animals. After the appearance of cancer lesions in the skin of all the animals (approx. in 60 days), they were grouped into three groups. The first group was the negative control, where no treatment was given to cure the cancer. In the second group, 500 mg/kg body weight of AML extract was given orally, while in the third group, Doxorubicin chemotherapeutic drug treatment (10 mg/kg BW) was given to cure the tumour. The increase/decrease in size of the tumour was measured in each group up to 120 days.

## 3. Results

### 3.1. Determination of Non-Toxic Dose of Argemone mexicana Leaves (AML) Extract

After evaluation of the parameters including haematology, body weight loss, and physical attributes in the animals across the groups, it was observed that the 1000 mg/kg BW of the dose was toxic for the animals and caused subacute toxicity, while the 100 mg/kg BW, 250 mg/kg BW and 500 mg/kg BW doses were safe for the animals. 

#### 3.1.1. Physical Attributes 

Some of the physical features of toxicity, observed in the animals across the groups, are given in Table 1. The animals of the 1000 mg/kg BW group were found to show the physical attributes of toxicity in comparison to other groups, which was considered subacute toxicity caused by AML extract.

#### 3.1.2. Haematological Parameter

**Figure 1:**
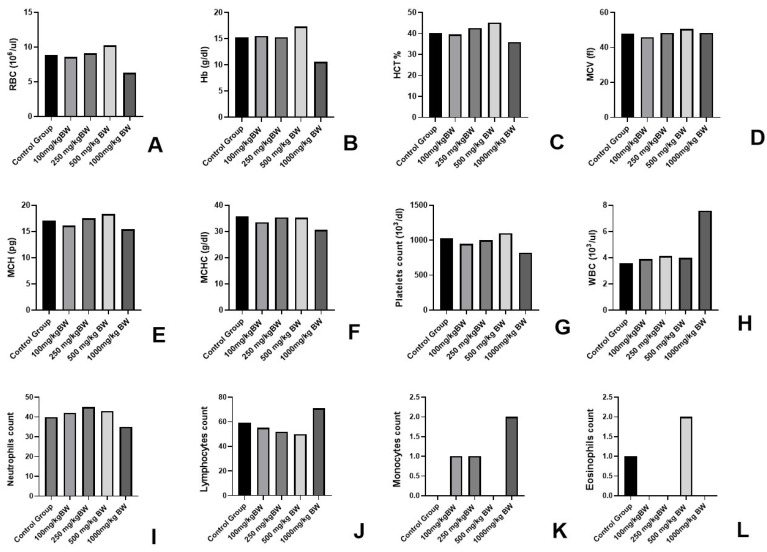
Haematological parameters in AML fed groups of experimental animals for determination of non-toxic dose (**A**–**L**). (**A**) RBC count, (**B**) haemoglobin, (**C**) HCT%, (**D**) MCV, (**E**) MCH, (**F**) MCHC, (**G**) platelets count, (**H**) WBC, (**I**) neutrophil count, (**J**) lymphocyte count, (**K**) monocyte count, and (**L**) eosinophils count from the blood samples of experimental animals.

In the haematological parameters, a decrease in WBC, RBC, and Hb values of the 1000 mg/kg BW group (Figure 1) indicated the toxic effect of the given dose in the animals. In other groups, no toxic effect was observed. An increase of 4–5% was observed in the 100 mg/kg BW, 250 mg/kg BW, and 500 mg/kg BW groups in parameters including RBC, Hb, MCV, MCH and platelet count.

#### 3.1.3. Body Weight

The body weights of mice in all the groups ranged 25–32 g at day 0 of the experiment. An average increase in body weight of 19.4% was noted in the control group animals after 21 days. The average increase in weight was observed to be 19.5% in 100 mg/kg BW, 22.8% in 250 mg/kg BW and 21.56% in 500 mg/kg BW of the AML-fed groups. However, a significant decrease (*p* > 0.001) in average body weight of −43.23% was found in the 1000 mg/kg BW group after 21 days of feeding the AML extract in comparison to the control group. This indicated the toxic effect of the 1000 mg/kg BW dose of AML extract (Table 2). 

### 3.2. Determination of Skin Cancer Preventive Activity of Argemone mexicana Leaves (AML) Extract

#### 3.2.1. Effect on Body Weight

**Figure 2:**
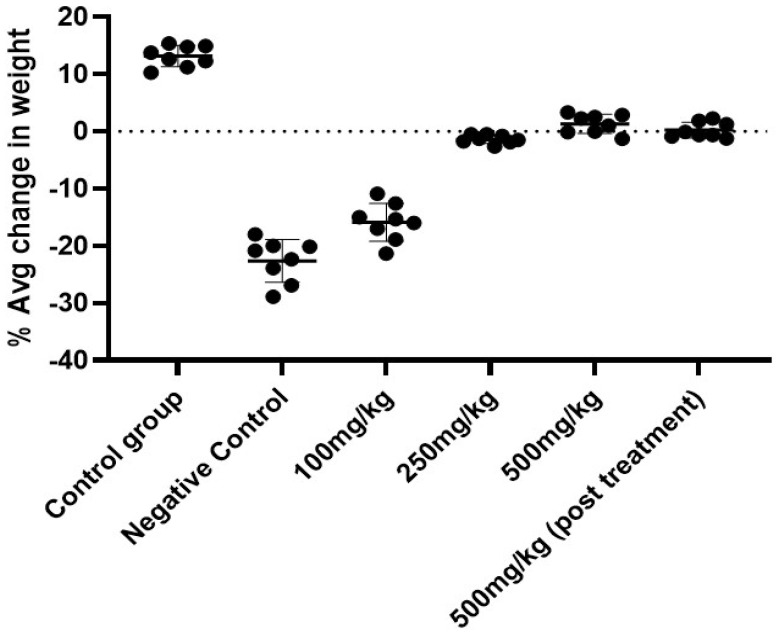
Percentage of weight change in cancer-induced experimental animals on the 60th day of the experiment.

The change in body weight was found in a dose-dependent manner in comparison to the negative group where a drastic decrease in weight could be noted due to the induction of cancer. On the 60th day, where an average weight gain in the control vehicle group was 13.8%, there was an average loss of weight in the 100 mg/kg BW (−15.93%), 250 mg/kg BW (−5%), 500 mg/kg BW (0.015%) and 500 mg/kg BW (P.T) (−1.2%) groups. The weight loss in the negative control group was found to be significantly reduced to −18.63% (*p* < 0.001) (Table 3; Figure 2). The 500 mg/kg BW group resisted the weight loss due to the effect of the AML extract.

#### 3.2.2. Effect on Tumour Volume 

**Figure 3:**
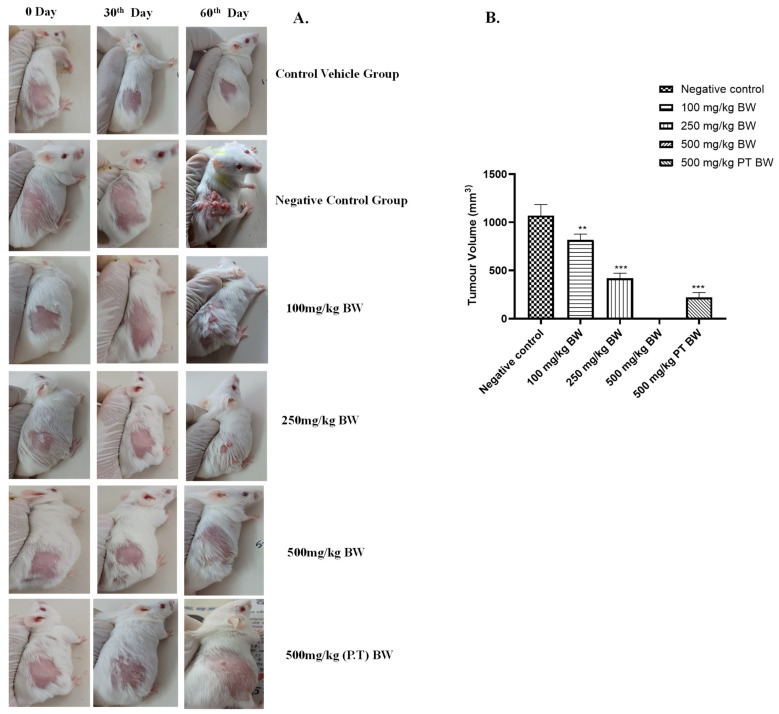
Prevention of cancer induction by AML extract. (**A**) The gross tumour appearance and variation in sizes amongst the group on the 30th and 60th day of post-cancer induction. The highest tumour load was observed in the negative control group, and there was no tumour growth or lesion in the 500 mg/kg BW group. (**B**) The tumour volume was found to be significantly reduced (*p* < 0.01) in the 100 mg/kg BW group, while the 250 mg/kg BW and 500 mg/kg BW groups (P.T) were found to be highly significant statistically *p* < 0.001 in comparison to the negative control group. No tumour growth in the 500 mg/kg body weight group was observed, indicating an extremely significant effect at this dose. In the figure, values are indicated as ** *p* < 0.01 and *** *p* < 0.001.

With the increase in time duration and exposure of DMBA/TPA, the size and burden of the tumour increased in each individual animal. The tumour size was also found to be reduced in a dose-dependent manner in the treatment groups. After 60 days, the tumour volume was found to be reduced significantly (*p* < 0.01) with an average decrease of 30% in the 100 mg/kg BW group. A significant reduction (*p* < 0.001) with an average decrease of 50–55% in the 250 mg/kg and 500 mg/kg BW (P.T) groups was observed with respect to the negative control group. There was a pre-cancerous lesion observed in 1–2 animals of the 500 mg/kg BW group, but no papule growth was found until the 60th day of the experiment, which showed the best cancer-preventive activity of AML extract (Figure 3).

#### 3.2.3. Cancer Induction in Mice

**Figure 4:**
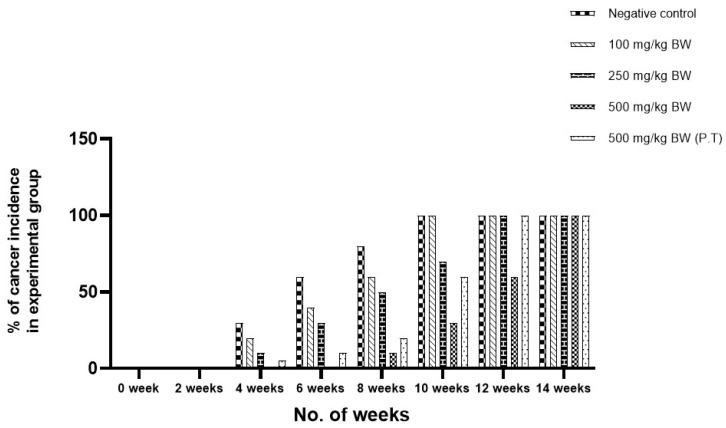
Percentage of cancer incidence in experimental animal groups using DMBA/TPA method. The observations have been made on the basis of occurrence of pre-cancerous lesions in the groups.

The process of cancer induction was started with the visible lesions on the shaved patch of skin which was painted by DMBA/TPA. Later, the gross papule formation was observed on the skin surface. The pre-cancerous lesion started to occur after ±4 weeks of giving treatment of DMBA/TPA in some animal groups. The appearance of gross papule formation was noted after 42 days of DMBA/TPA application in the negative control group. Some animals of the 100 mg/kg BW group, 250 mg/kg BW group, and 500 mg/kg (P.T) BW group also showed pre-cancerous lesions (% of animals is given in Figure 4). The pre-cancerous lesions in the 500 mg/kg BW group were observed after 8 weeks (±56 days) in comparison to the negative control group. The 500 mg/kg BW group showed pre-cancerous lesions after ±32 days of the appearance of cancer lesions as compared to the negative control group. A difference of ±15 days of cancer induction was observed in the 500 mg/kg pre- and post-treated group, which showed the cancer-preventive activity of the AML extracts (Figure 4). The negative control group as well as the 100 mg/kg BW, 250 mg/kg BW, and 500 mg/kg BW (P.T) groups started showing visible lesions 4 weeks after cancer induction, which then transformed into cancerous papilloma afterward. In comparison to the negative control group, only ~5% of cancer incidence was found in the 500 mg/kg BW group at the 8th week. The lesions converted into gross papules were observed in 40% (negative control group), 30% (100 mg/kg BW), 15% (250 mg/kg BW) and 5% (500 mg/kg BW (P.T)) of animals in the 4th week of the experiment. In the 10th week, the negative control group and 100 mg/kg BW group showed 100% induction of cancer in all animals, whereas ~75% incidence was rated in the 250 mg/kg BW group, 30% incidence was rated in the 500 mg/kg BW group, and 60% incidence was rated in the 500 mg/kg BW group (P.T). At the 14th week, 100% occurrence of cancer was found in all groups of animals.

#### 3.2.4. Effect on Histopathological Parameters of Skin Cancer Tissue

**Figure 5:**
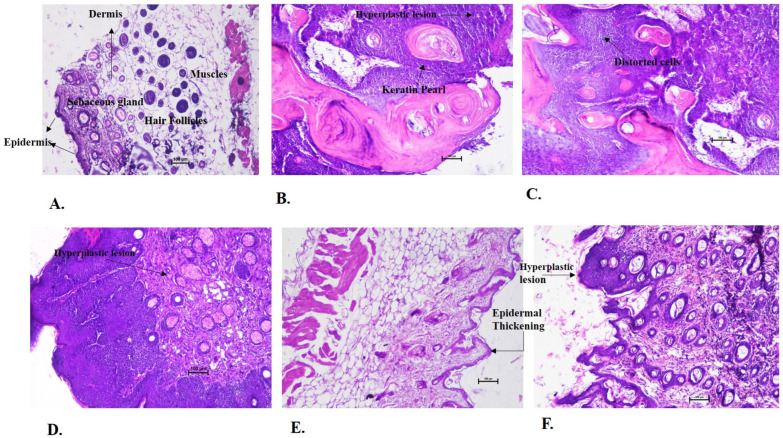
Histopathological examination of skin tissue from the sacrificed mice of all experimental groups. (**A**) The normal skin from the control vehicle group: all the layers of the skin including the epidermis, dermis, and hypodermis in their normal condition along with the hair follicles, and sebaceous gland, which are indicated with the help of arrows. The negative control group (**B**) showed highly distorted cells, hyperplastic lesions along with the formation of keratin pearls (indicated by arrow), large, dark coloured and uneven shaped nuclei indicated the squamous cell carcinoma. The 100 mg/kg BW group (**C**) showed similar structures as of (**B**), the formation of keratin pearls, and distorted cell arrangements as specified with the help of arrows, indicating the cancer formation. The 250 mg/kg BW group (**D**) showed a mixture of disarranged cells and normal cells. Hyperplastic lesions were observed along with benign types of cells (indicated by arrow). (**E**) The 500 mg/kg BW group showed a similar structures to the control vehicle group but with a slight thickening in the epidermis layer as labelled with the help of arrows. Whereas in the (**F**), the disorderly arranged cells were observed at some sights. Hyperplastic thickening was also noted (as labelled). Scale bar = 100 µm.

The histopathological sections of the skin tissues of all experimental groups were observed, and examination of the control vehicle group revealed the normal cells of the skin (Figure 5A). The layers of skin had a well-distinguished epidermis, dermis, and hypodermis layer along with the muscle layers. Sebaceous glands and hair follicles along with the fatty layer stained distinctly. In contrast, in the negative control group (Figure 5B), a thickened hyperplastic epidermis and immature stratum corneum were observed. The formation of keratin pearls along with a large number of distortedly arranged cells with enlarged nuclei indicating their transformation into benign cells were noted. The nucleus was enlarged and dark, and cells were in an uneven and bizarre formation, indicating the severe growth of cancer and the conversion of normal cells into cancerous cells. The papilloma growing in the outwardly direction and the cancer was identified as squamous cell carcinoma. Similar features were observed in the 100 mg/kg BW group (Figure 5C). In the 250 mg/kg BW group (Figure 5D), the hyperplastic lesions were observed along with the thickened epidermis. The cells were found distorted and unorderly arranged, but at the same time, normal cells were also present. A higher percentage of normal cells were observed in comparison to the distorted cells. The nucleus of some cells was enlarged, indicating their transformation into benign cells. 

The 500 mg/kg BW group (Figure 5E) appeared to have a similar histopathological finding as of the normal skin, but a slight thickening in the epidermis layer was identified. The 500 mg/kg (P.T) BW (Figure 5F) group showed hyperplastic thickening of the epidermis layer along with disorderly arranged cells at some sites, as shown in Figure 5F. The experiment concluded the effect of AML extract in the prevention of cancer formation and its preventive effect in the conversion of normal cells to cancerous cells in a dose-dependent manner. 

#### 3.2.5. Effect on Haematological Parameters

**Figure 6:**
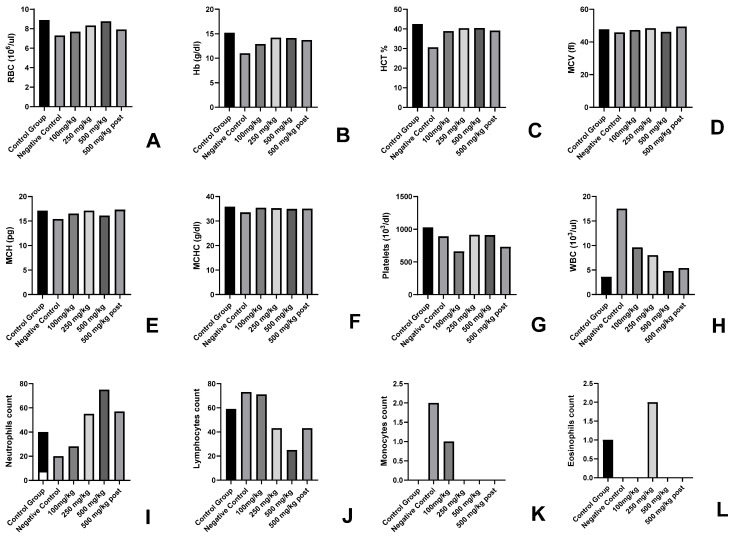
Effect of AML extract of haematology of cancer-induced experimental animals (**A**–**L**). Where (**A)** represents the RBC count, (**B**) haemoglobin, (**C**) HCT%, (**D**) MCV, (**E**) MCH, (**F**) MCHC, (**G**) platelets count (**H**) WBC, (**I**) neutrophil count, (**J**) lymphocyte count, (**K**) monocyte count, and (**L**) eosinophils count from the blood samples of experimental animals.

In the haematological parameters, a decrease was observed in the values of WBC, RBC, Hb, etc. (Figure 6) in the negative control group animals due to cancer load. Alterations in these parameters were observed in the 100 mg/kg BW, 250 mg/kg BW, and 500 mg/kg BW (P.T) groups. The values were found to be normal in the 500 mg/kg BW group. The haematological parameters could be found correlated to the cancer load in the animals and cancer preventive activity of the AML extract in a dose-dependent manner (Figure 6).

#### 3.2.6. Effect on Weight and Size of Liver and Spleen

**Figure 7:**
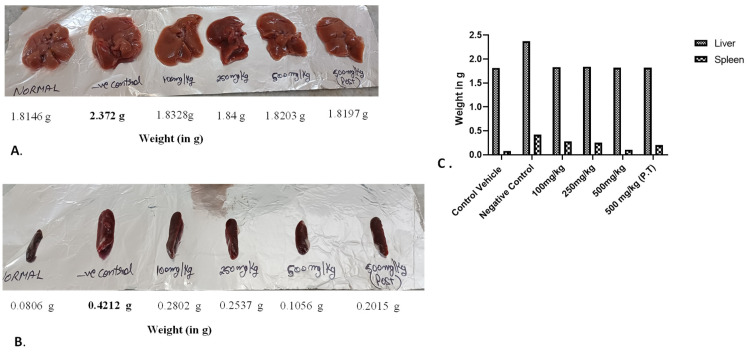
Size and weight of (**A**) liver, (**B**) the spleen, (**C**) average weight of organ from the sacrificed experimental animals on the 60th day of the experiment. It was observed that (**A**) the liver in the negative control group is enlarged whereas it was found to be normal in other groups. In the case of the spleen, (**B**) splenomegaly was observed in the negative control group, while the other groups exhibited a slight increase in the size of the spleens. The spleen of the 500 mg/kg BW group was observed to be normal in size.

The negative control group had an increase in the size and weight of the liver as compare to other groups, as indicated in the Figure 7. In the case of the spleen, normal weight and size were found in the 500 mg/kg group, which was comparable to the control vehicle group, indicating the effect of AML extract on the cancer induction. In the negative control group, as well as the 100 mg/kg BW, 250 mg/kg BW, and 500 mg/kg BW groups (P.T), the spleen was found to exhibit splenomegaly due to the increased production of WBCs under the influence of cancer induction (Figure 7). 

#### 3.2.7. Effect on Inflammatory Cytokine TNF-α Concentration in Serum 

**Figure 8:**
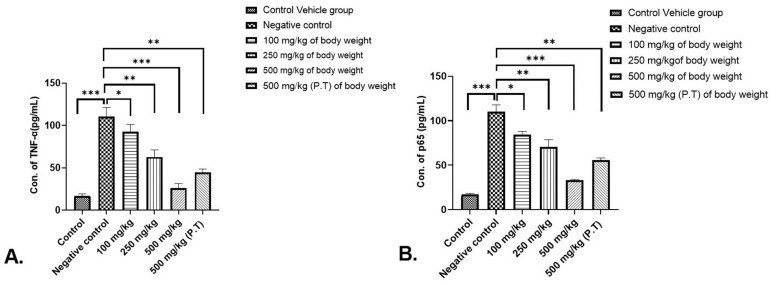
The alteration in the pattern of (**A**) TNF-α expression and (**B**) p65 subunit of NF-κB signalling pathway in the serum where the concentration of TNF-α was found to be downregulated in the 500 mg/kg BW group significantly (*p* < 0.001). The values are represented in ±SEM. * *p* < 0.05, ** *p* < 0.01 and *** *p* < 0.001. The results were calculated using one-way ANOVA followed by Dunnett’s test.

The concentration of TNF-α was found to be increased significantly in the negative control group (*p* < 0.001) due to cancer induction in comparison to the control vehicle group. Also on the other hand, there was a significant decrease in TNF-α concentration in 500 mg/kg BW group (*p* < 0.001) vs. the negative control group. The concentrations were also found to be highly significant (*p* < 0.01) in 500 mg/kg BW (P.T) and in 250 mg/kg BW. While in 100 mg/kg BW group a significant (*p* < 0.05) decrease was observed in comparison to the negative control group (Figure 8A). 

#### 3.2.8. Effect on p65 Subunit Concentration of NF-κB Signalling Pathway in Serum 

A similar trend as of TNF-α was found in the p65 concentration of NF-κB signalling pathway where *p* < 0.001 in the negative control group vs. control vehicle group. The p65 concentration was found to decrease significantly for 500 mg/kg BW (*p* < 0.001) vs. the negative control group. A significant reduction in the concentration was also found in 500 mg/kg BW (P.T.) as well as 250 mg/kg BW (*p* < 0.01), and 100 mg/kg BW (*p* < 0.05) in comparison to the negative control group (see Figure 8B). 

### 3.3. Determination of Skin Cancer Treatment Activity of Argemone mexicana Leaves (AML) Extract

To compare the effect of AML extract with the standard chemotherapeutic agent, the treatment was started after the induction of skin cancer with 500 mg/kg BW of AML extract in one group and 10 mg/kg BW of Doxorubicin in the second group. But neither the AML extract nor the Doxorubicin were able to cure the skin cancer in the experimental animals. No alteration or difference in the cancer reduction was observed under the influence of both treatments.

## 4. Discussion

One out of every six recorded fatalities worldwide is caused by some sort of malignancy. It is concerning because it has shown that the incidence of tumours and cancers are increasing over time. One of them, skin cancer, is a fatal cancer that is common in the United States [1]. Since the skin is the largest organ in the body and cancer often begins in the epidermis, DNA damage brought on by oxidative stress may be a contributing factor [21]. Chemotherapy, radiotherapy, surgery, and oral medications make up the foundation of a patient’s treatment, but the situation never seems to stabilise [22]. Exploration of the benefits of the plant-derived phytoconstituents for chemo-preventive activity has been researched for skin cancer. Similar shreds of evidence could be derived from researchers like Klos and Chlubek (2022), who gave a comprehensive study on plant terpenoids for anti-melanoma and their synergistic action of combining terpenoids with other substances [23]. *Argemone mexicana* Linn (AM) is one such plant that possess antimicrobial [24], cytotoxic [25], antifungal [26] and anti-inflammatory activity [27]. Its pharmacological activity was recently explored by Jaiswal et al. (2023) [28]. It has been experimented on for its anticancerous properties by different researchers including Chang et al. (2003), Singh et al. (2016) and Prabhakaran et al. (2017) for its different plant parts, and they found significant results [29,30,31]. Exploring the anticancerous activity of AM is still an area for potential research, but it draws suspicion as it is well known for its toxic nature and notorious for the case of epidemic dropsy [32]. Because of the presence of Sanguinarine and its analogues, seeds and flowers are toxic, but the leaves of *Argemone mexicana* do not contain these components and are non-toxic; thus, they are utilised in the present study. A study conducted in clinical trials in Mali and Switzerland also gave evidence supporting the use of *Argemone mexican* plant extract/sap against malaria in humans [9]. The TNF-α, nuclear factor-*k*B (NF-κB), *STAT3*, *AKT*, and *COX-2* are linked with different stages of cancer progression and reported to regulate cancer proliferation, apoptosis, invasion, metastasis, and angiogenesis. This highlights the important role of such factors in cancer prevention. In 2003, Ueda and Richmond reported on the expression of inflammatory cytokines, NF-κB pathway stimulation, and other immunological events that lead to inflammation and eventually to cancer [33]. 

In the present study, the ethanolic extract of *Argemone mexicana* Linn leaves which was found to possess the best anticancer activity in the in vitro analysis (unpublished data) was employed for the in vivo study in a mice model. The non-toxic dose determination was made with the different doses, and the dose of 1000 mg/kg body weight was found to be toxic as it induced lethargy, weight loss, and alterations in the haematological findings of the experimental animals. The average weight loss after a dose of 1000 mg/kg body weight was −43.3%, which denoted the toxicity. On the other hand, a slight increase of 22.8% and 21.56% in body weight was observed with the 250 mg/kg and 500 mg/kg doses, respectively. The immunomodulatory property of *Argemone mexicana* Linn was reported by Goel (2020) and Perez et al. (2023) [27,34], which could be accountable for the weight gain in our studies. It is also supported by the 4–5% increase in RBC, Hb, MCV, MCH, and platelet counts at 250 mg/kg BW and 500 mg/kg BW. The 1000 mg/kg body weight dose caused subacute toxicity, and no causality has been observed in the animals. The study of the chemo-preventive potential of the AML extract was carried out in DMBA/TPA-induced skin cancer in a mice model along with the doses 100 mg/kg, 250 mg/kg and 500 mg/kg body weight. The mice model by the DMBA/TPA method has been employed for the skin cancer studies by researchers including Manoharan and Sharmila where they used rosmarinic acid for its anticancer activity [11]. The animals were examined through various parameters including change in weight, incident of cancer induction, blood parameters, histopathology, etc. In the present study, weight loss was directly related with the severity of cancer. Similar findings of weight loss with cancer were reported in previous studies [10,11]. 

The DMBA/TPA induction resulted in 100% tumour in the negative control group. The tumour volume and burden could also be correlated with the loss in weight. The average tumour burden in the negative control group was maximum with the volume of 1089.69 mm^3^. The tumour volume was reduced in the AML extract groups. It was found to be 895.89 mm^3^ in the 100 mg/kg BW group, 423.96 mm^3^ in the 250 mg/kg BW group and 296.89 mm^3^ in the 500 mg/kg BW (P.T) group on the 60^th^ day of the experiment, and only two animals showed pre-cancerous lesions in the 500 mg/kg BW group. The animals that developed the pre-cancerous lesions were stopped with the DMBA/TPA treatment, but the treatment of DMBA/TPA was continued in other animals up to the time when they started developing cancer lesions. The experiment clearly indicated the extended time interval of cancer induction in the 500 mg/kg BW group of AML. A very clear-cut difference of ±32 days was observed in between the negative control group and 500mg/kg BW group in the development of cancer in all the animals in both the groups. A difference of ±15 days of cancer induction could be observed in the 500 mg/kg pre- and post-treated group, which showed the cancer-preventive activity of the AML extracts. A similar experiment was quoted by Sharmila and Mahoharan (2012), where pretreatment of rosmarinic acid prevented the skin cancer induction in a mice model [10].

In the present study, haematological blood parameters were also found to be disturbed in different groups. But the maximum decrease in RBC and other haematological parameters was observed in the negative control group, and the best results could be found in the 500 mg/kg group, which supported the anticancerous activity of the group at the haematological level. The blood parameters like decrease in the number of RBC and increase in WBC act as a marker for disease, as was also quoted by Connell et al. [35]. 

Histopathology revealed the occurrence of distorted cells with enlarged nuclei, the formation of keratin pearls, and hyperplastic lesions in the negative control group, which clearly evidenced the formation of squamous cell carcinoma in the animals. These pathological findings were supported with the histopathological features of non-melanoma skin cancer as described by Chen et al. (2009) [36] and Paolino et al. (2017) [37]. The 100 mg/kg BW group showed similar features to the negative control group, which indicated that the AML extract had a non-significant effect on cancer prevention in the animals at this dose. On the other hand, the presence of a greater number of normal cells than the distorted hyperplastic cells and a few of them on the verge of turning cancerous highlighted the cancer-preventive effect of the 250 mg/kg dose of AML. In the 500 mg/kg BW group, the histopathology was found to be similar to that of the normal skin but with a bit of thickening and abnormality in the epidermal layer, which could be correlated to the occurrence of pre-cancerous lesions in the animal. The 500 mg/kg (P.T) BW of the group showed the presence of hyperplasia at certain sites, and the other part of the tissue was found to be normal. The pretreatment at the 500 mg/kg BW dose was found to be better than the post-treatment 500 mg/kg group in resisting the cancer induction. This could be supported by the fact that the Ayurveda always focuses on prevention rather than cure. The results were compared and correlated with the studies where geraniol, an acyclic monoterpene alcohol, and rosmarinic acid prevented the formation of DMBA-induced skin cancer in a mice model [10,11].

The effect of the dose of AML extract could also be seen on the weight of the organs extracted from the sacrificed animals. Splenomegaly could be observed in the spleen of negative control animals due to the increased no. of WBCs under the influence of cancer induction. The variation in sizes of the spleens could be noticed in a dose-dependent manner where the weight of the spleens in the 500 mg/kg BW group was almost similar to the weight of spleens in the control vehicle group. The immunomodulatory response of aqueous extract of AML was also reported earlier [27]. The liver could only be seen enlarged in the negative control group and was similar to normal in the other groups. The findings were in synchronisation with the severity of cancer induction amongst the groups.

The TNF-α is a pro-inflammatory cytokine involved in a tumorigenic role [38]. The DMBA/TPA carcinogenesis process involves a TNF-α mediated increase in nuclear translocation of NF-κB (A transcription factor), which facilitates the survival and proliferation of neoplastic cells [39]. In the present study, the TNF-α concentration was found to be decreased significantly in the 500 mg/kg BW group vs. the negative control group (*p* < 0.001), whereas the concentration of TNF-α was found to decrease significantly (*p* > 0.01) in the 250 mg/kg BW group, 500 mg/kg BW group and (*p* < 0.05) for the 100 mg/kg BW group. The decreased TNF-α concentration in the serum of the AML extract treatment groups indicated the anti-inflammatory potential of the extract and accounts for its anticancerous potential. 

An inducible transcription factor called nuclear factor-kappa B (NF-κB) controls the expression of several genes related to the immune response. Increased NF-κB activation could result from disruptions in upstream signalling networks like PI3K/Akt and Ras/Raf. Because of the inflammatory milieu, constitutive NF-κB activation is present in a considerable number of human malignancies. Since tumour regression is typically the result of NF-κB suppression in tumour cells, the NF-κB pathway is a prospective target for therapeutic intervention [40,41]. As indicated in this study, in the NF-κB signalling pathway, the concentration of the p65 subunit was found to be downregulated in the 500 mg/kg BW group significantly (*p* < 0.001), (*p* < 0.01) in the 250 mg/kg group and 500 mg/kg PT group and *p* < 0.05 in the 100 mg/kg group, as compared to the negative control group. In the other AML extract-treated groups also, the p65 concentration was reduced, indicating the role of AML extract in tumour suppression. This study confirms the blocking of the NF-κB inflammatory pathway for the production of different inflammatory cytokines as well as supports the above findings where cancer was prevented maximally by AML extract at 500 mg/kg BW of the dose in experimental animals.

As the treatment of AML extract as well as standard Doxorubicin drug did not show any curative effect, it was thus found to be insignificant. This again emphasises the better results of the pretreatment with AYUSH drugs. 

## 5. Conclusions

Skin cancer is one of the most dreadful cancers and a leading cause of death in the world. Extensive research has been conducted in the field of ethnopharmacological exploration to investigate the therapeutic potential of herbs and medicinal plants against cancer. The results have shown encouraging findings. The phytochemicals isolated and explored for anticancer activities are mainly used as adjuvants along with chemotherapeutic drugs to support the health of the patients. The experiments included in this study demonstrated the cancer-preventive effect of *Argemone mexicana* Linn leaves against skin cancer-induced mice model. Due to its synergistic effects, the crude ethanolic extract was chosen for the investigation instead of the isolated phytoconstituents, as it is known to produce better outcomes. It could be concluded that the best dose at which the anticancerous activity was found is 500 mg/kg BW of AML extract. The difference in the cancer progression and occurrence was also found in a dose-dependent manner. Even though the study involved the extract’s first screening and demonstrated its anticancer effects in a mouse model, it had drawbacks, such as the ability to chemically induce cancer. The tumour induced was also of animal origin instead of human origin. The chemically induced models are inexpensive, but xenograft and allografts model hold better opportunities for having an insight of drug action as they mimic the human tumour environment. In order to properly interpret the findings and comprehend the mechanism in other models, the study maintains the belief that it should be investigated in other animal models, such as patient-derived xenograft animal models. The ethanolic extract from *Argemone mexicana* Linn leaves could be explored as a novel therapeutic treatment for skin cancer and potentially slowing down its progression. In order to explore the *Argemone mexicana* Linn leaves extract in clinical trials, to bridge the gap for utility, a thorough investigation is required. 

## Figures and Tables

**Table 1:** Physical features in experimental animals for determination of non-toxic dose of *Argemone mexicana* Linn leaves extract.

Groups	100 mg/kg BW	250 mg/kg BW	500 mg/kg BW	1000 mg/kg BW
Lethargy	-	-	-	++
Nausea	-	-	-	+
Dullness/Loss in fur	-	-	-	++
Loose Motion/Loose stool	-	-	-	++
Yellow eyes	-	-	-	+
Fatality	-	-	-	-

The signs represent (++) as Moderate, (+) as Mild and (-) as Nil.

**Table 2:** The average change in weight of experimental animals for determination of maximum non-toxic dose where *** *p* < 0.001 is a significant decrease in weight in comparison to the control group. The values are expressed as mean ± SD for 8 animals. The results were calculated using one-way ANOVA followed by Dunnett’s test.

Type of Group	Average Body Weight at Day 0 (in g)	Average Body Weight at Day 21 (in g)	% Average Increase
Control group	33.9 ± 3.9	40.1 ± 1.6	19.4%
100 mg/kg BW	34.6 ± 2.9	41.4 ± 1.9	19.5%
250 mg/kg BW	35.6 ± 1.2	43.7 ± 3.7	22%
500 mg/kg BW	35.0 ± 1.3	42.2 ± 2.1	18%
1000 mg/kg BW	37.5 ± 2.6	21.2 ± 3.6 ***	−43.23%

**Table 3:** The average change in body weight in cancer-induced animals along with the given treatment of AML where a significant decrease *p* < 0.001) in the negative control group was observed. The values are expressed as means ± SD for 8 animals. Values indicating a = *p* < 0.001, b = *p* < 0.05, and c = *p* < 0.05 were found to be statistically significant. The results were calculated using one-way ANOVA followed by Dunnett’s test.

Type of Group	Body Weight at Day 0 (in g)	Body Weight at Day 21 (in g)	Body Weight at Day 35 (in g)	Body Weight at Day 49 (in g)	Body Weight at Day 60 (in g)
Control group	33.9 ± 3.6	33.15 ± 1.2	36.2 ± 2.3	36.6 ± 1.6	38.9 ± 2.6 ^c^
Negative control	39.4 ± 2.3	38.4 ± 6.6	37.3 ± 9.3	35.6 ± 8.3	32.3 ± 6.9 ^a^
100 mg/kg	35.9 ± 5.9	36.7 ± 4.9	39.9 ± 6.3	31.4 ± 5.6	31.2 ± 6.3 ^a^
250 mg/kg	34.5 ± 2.3	35.73 ± 1.6	39.9 ± 5.9	35.5 ± 3.9	32.8 ± 6.9 ^b^
500 mg/kg	39.2 ± 6.9	38.9 ± 3.2	39.6 ± 5.9	39.9 ± 2.1	38.75 ± 6.3 ^b^
500 mg/kg (post-treatment)	36.9 ± 2.3	35.6 ± 1.3	35.9 ± 9.3	34.2 ± 6.3	34.5 ± 4.3 ^b^

## Data Availability

Data are contained within the article.

## Abbreviations

DMBA	7,12-dimethylbenz[a]-anthracene
TPA	*12-O-Tetradecanoylphorbol-13-acetate*
B.W	Body Weight
AML	*Argemone mexicana* leaves
TNF-α	Tumour Necrosis Factor-α
NF-κB	Nuclear Factor Kappa-B
IKK	IκB Kinase
NEMO	NF-κB essential modulator
DPX	Dibutylphthalate Polystyrene Xylene
NBF	Neutral Buffer Formalin
SD	Standard Deviation
